# Fast emerging insecticide resistance in *Aedes albopictus* in Guangzhou, China: Alarm to the dengue epidemic

**DOI:** 10.1371/journal.pntd.0007665

**Published:** 2019-09-16

**Authors:** Xinghua Su, Yijia Guo, Jielin Deng, Jiabao Xu, Guofa Zhou, Tengfei Zhou, Yiji Li, Daibin Zhong, Ling Kong, Xiaoming Wang, Min Liu, Kun Wu, Guiyun Yan, Xiao-Guang Chen

**Affiliations:** 1 Department of Pathogen Biology, School of Public Health, Southern Medical University, Guangzhou, China; 2 Program in Public Health, University of California, Irvine, Irvine, CA, United States of America; North Carolina State University, UNITED STATES

## Abstract

Dengue is one of the most serious mosquito-borne infectious diseases in the world. *Aedes albopictus* is the most invasive mosquito and one of the primary vectors of dengue. Vector control using insecticides is the only viable strategy to prevent dengue virus transmission. In Guangzhou, after the 2014 pandemic, massive insecticides have been implemented. Massive insecticide use may lead to the development of resistance, but few reports are available on the status of insecticide resistance in Guangzhou after 2014. In this study, *Ae*. *albopictus* were collected from four districts with varied dengue virus transmission intensity in Guangzhou from 2015 to 2017. Adult *Ae*. *albopictus* insecticide susceptibility to deltamethrin (0.03%), permethrin(0.25%), DDT(4%), malathion (0.8%) and bendiocarb (0.1%) was determined by the standard WHO tube test, and larval resistance bioassays were conducted using temephos, *Bacillus thuringiensis israelensis* (*Bti*), pyriproxyfen (PPF) and hexaflumuron. Mutations at the voltage-gated sodium channel (VGSC) gene and acetylcholinesterase (AChE) gene were analyzed. The effect of cytochrome P450s on the resistance of *Ae*. *albopictus* to deltamethrin was tested using the synergistic agent piperonyl butoxide (PBO). The results showed that *Ae*. *albopictus* populations have rapidly developed very high resistances to multiple commonly used insecticides at all study areas except malathion, *Bti* and hexaflumuron. We found 1534 codon mutations in the VGSC gene that were significantly correlated with the resistance to pyrethroids and DDT, and 11 synonymous mutations were also found in the gene. The resistance to deltamethrin can be significantly reduced by PBO but may generated cross-resistance to PPF. Fast emerging resistance in *Ae*. *albopictus* may affect mosquito management and threaten the prevention and control of dengue, similar to the resistance in *Anopheles* mosquitoes has prevented the elimination of malaria and call for timely and guided insecticide management.

## Author summary

Guangzhou is the most epidemic area of dengue in China. Massive insecticides have been used to control the vector mosquito *Ae*. *albopictus*, as no specific vaccines are available for dengue. Regular monitoring of insecticide susceptibility is essential for insecticide resistance management. In this study, the insecticide resistances of *Ae*. *albopictus* in Guangzhou were comparatively analyzed from 2015 to 2017. The results displayed that *Ae*. *albopictus* had rapidly generated high resistance to the most commonly used adult insecticide pyrethroid (deltamethrin and permethrin) and larvicide organophosphate (temephos). The combination of malathion for adult mosquitoes and *Bti* or hexaflumuron for larvae might be a better choice for vector control. Resistance to deltamethrin can be significantly reduced by PBO but may generated cross-resistance to PPF. F1534S and F1534L mutations in the VGSC gene were significantly correlated with resistance to pyrethroids. This study indicated that the insecticide resistances had been generated in *Ae*. *albopictus* in Guangzhou which was correlated with the dengue epidemic responses.

## Introduction

Dengue is one of the most rapidly spreading mosquito-borne diseases in the world. Currently, 3.9 billion people in 128 countries or regions are at risk of dengue fever [1–3]. Guangzhou, the largest city in southern China and the capital of Guangdong Province, has become the epicenter of dengue outbreaks in China. In Guangzhou, the number of dengue cases accounted for 50% of the national incidence between 1978–2011 and an epidemic had occurred once every 3–4 years since the 1990s [4, 5]. In particular in 2014, a pandemic of dengue broke out in Guangzhou with more than 37,000 cases reported [6].

*Aedes albopictus* is the most invasive mosquito and is widely distributed in China, from Hainan in the south to Dalian in the north, while *Aedes aegypti* is only distributed in Hainan, Yunnan and a small area of the southernmost part of Guangdong Province [7]. *Ae*. *albopictus* is the main vector for dengue virus in China and in Guangzhou *Ae*. *albopictus* is the sole vector of dengue virus [8, 9]. Currently, due to the lack of effective drugs and vaccines against dengue, vector management is the main strategy to prevent and control mosquito-borne diseases, including dengue [10–13]. In China, chemical control through the use of insecticides is one of the major tools for the control of vector mosquitoes [14, 15]. During the outbreak of dengue in Guangzhou in 2014, more than 27,000 kg of pyrethroids were used for ultralow-volume (ULV) spraying to control adult *Ae*. *albopictus*, and a large amount of temephos, an organophosphate larvicide, was used for larval control. Chemical insecticides were also frequently used for focal hot-spot control of sporadic dengue transmission in Guangzhou [16, 17]. At the same time, agricultural insecticide usage in rural areas and residential insecticide usage in the city affected the resistance of *Ae*. *albopictus* in Guangzhou, although insecticide use is greater in the public health field. The government also regularly organized the patriotic health campaign to clean up aquatic mosquito habitats in Guangzhou.

With the extensive use of insecticides, insecticide resistance has become a threat. Since 2014, resistance to some insecticides has been reported in *Ae*. *albopictus* in limited regions of Guangzhou. Li et al. reported that the *Ae*. *albopictus* adult population in Yuexiu had developed resistance to dichlorodiphenyltrichloroethane (DDT), and deltamethrin [18]. The reports of insecticide resistance in Guangzhou raise serious concerns about the efficacy of chemical insecticides against *Ae*. *albopictus* and the dengue transmission control policy in China. Current research on the resistance mechanism of *Ae*. *albopictus* mainly focuses on target-site insensitivity and increased metabolic detoxification of insecticides[19]. Non-synonymous mutations in the voltage-gated sodium channel (VGSC) gene that cause resistance to pyrethroids and DDT insecticides are known as knockdown resistance (*kdr*) [18, 20, 21] and mutations (*ace-1*) in the acetylcholinesterase (AChE) gene cause a resistance to carbamates and organophosphates [22, 23]. However Grigoraki et al. reported that the resistance of *Ae*. *albopictus* to temephos is associated with elevated carboxylesterases (CCEs) which is caused by up-regulation of CCEae3a gene [24], and no difference was detected between resistant and susceptible CCEae3a_aeg variants [25]. Detoxification pathways are very complex and can be divided into three major gene families, monooxygenases (P450s), carboxylesterases (COEs), and glutathione S-transferases (GSTs) [26]. P450s are related to pyrethroid resistance in *Ae*. *albopictus* [27].

Determine the insecticide resistance status and mechanisms of *Ae*. *albopictus* in Guangzhou is very important for local vector control. In this study, *Ae*. *albopictus* was collected from four districts in Guangzhou during 2015–2017 and the resistances to the currently used insecticides was comparatively analyzed through a series of experiments. The aim was to characterize the spatial distribution, temporal changes, and mechanism of insecticide resistance in Guangzhou, and provide guidance for monitoring and controlling vector mosquitoes and mosquito-borne diseases.

## Methods

### Study sites

**Fig 1:**
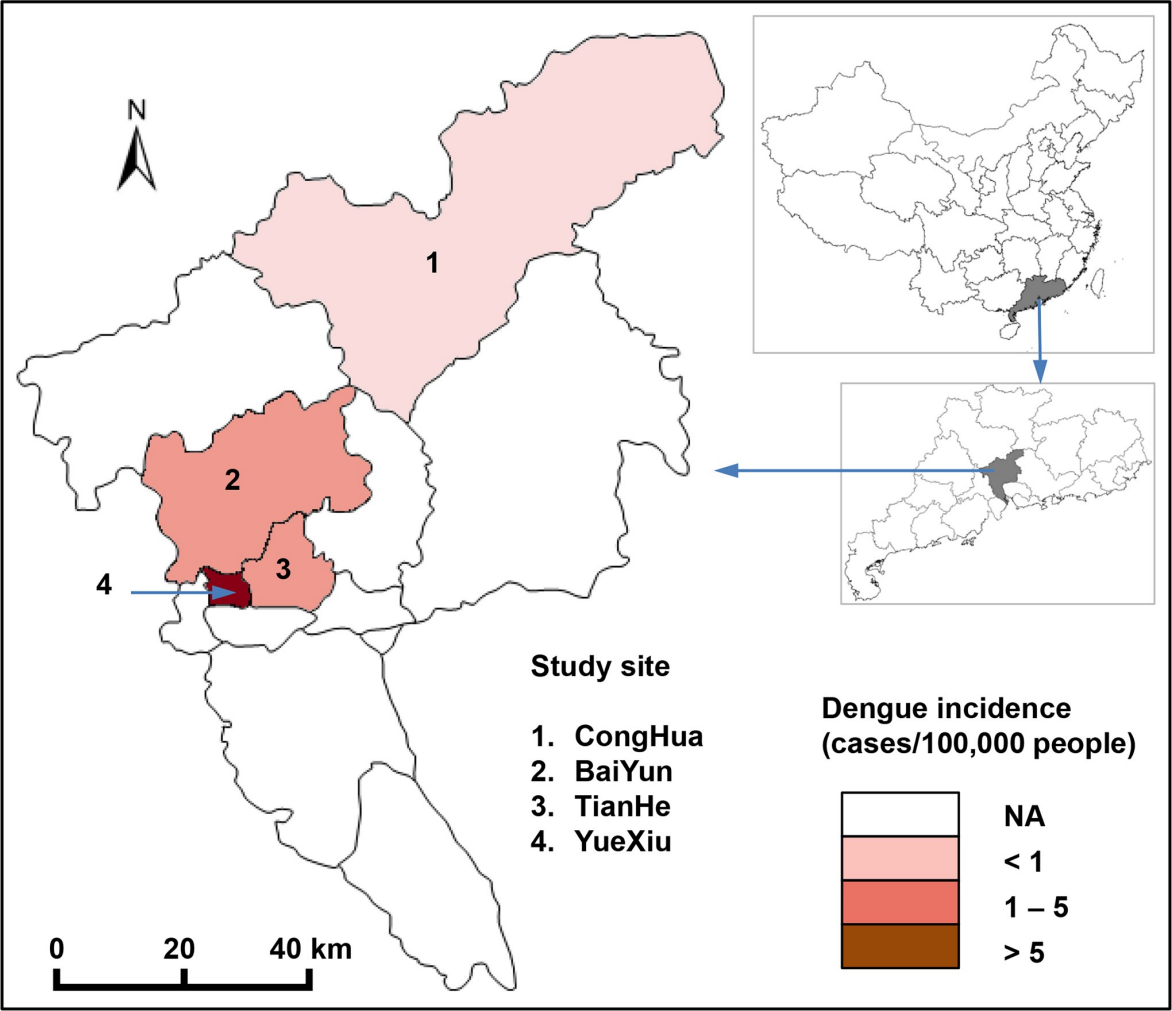
The study sites and corresponding dengue incidence rates in Guangzhou, China. The map was created using ArcGIS.

The study was conducted in four districts in Guangzhou, Guangdong Province, China, from 2015 to 2017: 1) Yuexiu district is located in the old downtown area, 2) Tianhe district is located in the new downtown area, 3) Baiyun district is located in the suburban area, and 4) Conghua district is located in the rural area. The reported dengue incidence varied among the four districts. The study sites and dengue incidence rates was marked in Fig 1, which was created by ArcGIS 10.2. The research site is a subtropical area with a monsoon climate. The annual average annual temperature is 20–22°C, the average relative humidity is 77%, and the annual rainfall is approximately 1720 mm.

### Mosquito strains and collection

The Foshan strain of *Ae*. *albopictus* was used as a control in this study, which was collected from Foshan City in 1983, and kept in the laboratory without insecticide exposure since then.

*Ae*. *albopictus* larvae were collected from three localities in each of the four districts, with representatives samples collected from parks, schools and residential areas, and all collection was done on public land (S1 Table). The larvae were housed in a steel tank with a size of 23 cm*29 cm*6.5 cm, and 1.5–2 L of dechlorinated tap water and small turtle food for feeding were added to the tank. Adult mosquitoes were housed in 20 cm*45 cm*30 cm yarn cages and fed with 10% glucose water. The female mosquitoes were bloodfed from an anesthetized mouse for spawning. The larvae were reared in the laboratory until adulthood. In the laboratory, the temperature was maintained at 26 ± 2°C, the relative humidity was 70 ± 10%, and the light: dark cycle was 14 h: 10 h. Non-blood-fed F1-generation female mosquitoes aged 3–5 days were used for the resistance test.

R24 is a laboratory resistant strain selected with deltamethrin for 24 generations from susceptible *Ae*. *albopictus* populations. Selection was performed by exposing each generation of fourth-stage larvae for 24 h to a 50% lethal concentration (LC_50_) of deltamethrin. The LC_50_ was determined by a larval bioassay. After 24 generations, the LC_50_ of deltamethrin increased from 0.001 mg/L to 0.033 mg/L.

### Adult resistance bioassays

The adult resistance bioassays were performed using five insecticides, including the four major classes of insecticides currently used which were recommended by WHO Pesticides Evaluation Scheme (WHOPES) [28], i.e., type II pyrethroid: deltamethrin; type I pyrethroid: permethrin; organophosphates: malathion; organochlorine: DDT; and carbamate: bendiocarb, following the standard WHO tube test protocol [29,30]. We used deltamethrin (0.03%), permethrin (0.25%), malathion (0.8%) and bendiocarb (0.1%) test for 1h and DDT (4%) test for 0.5h by the standard WHO tube test. Testing kits and insecticide-impregnated papers with standard diagnostic doses were provided by the Universiti Sains Malaysia, Penang, Malaysia. In each holding tube, 25 adult female mosquitoes were tested with five replicates of field mosquitoes and two replicates of controls. The number of adult mosquitoes knocked down was recoded every ten minutes and used to calculate the value of 50% knockdown times (KDT_50_). After 1 h of exposure, the mosquitoes were transferred to holding tubes and fed on a 10% sucrose solution for 24 h. Mortality was scored after 24 h of recovery to determine the susceptibility status. After the bioassay, the dead and live mosquitoes were separated and stored individually in 95% alcohol for subsequent DNA analysis.

### Adult synergistic test

The effect of cytochrome P450s on the resistance of *Ae*. *albopictus* to deltamethrin was tested using the synergistic agent piperonyl butoxide (PBO) following WHO guidelines [29,30]. Test paper with 4% PBO was prepared from 95% PBO (Yien Co. Ltd, Shanghai, China). Experiments on sets of 25 field-collected female mosquitoes were performed separately for 1) exposure to PBO alone for 1 h; 2) exposure to PBO for 1 h followed by exposure to deltamethrin for 1 h; and 3) exposure to deltamethrin alone for 1 h; and 4) control: no exposure to any agent. After the 1 h experiments, experimental mosquitoes were transferred to holding tubes, and mortality at 24 h was documented. This process was repeated five times.

### Larval resistance bioassays

Mosquito larval resistance bioassays were conducted using four insecticides which were recommended by WHOPES [31]: 1) organophosphate, temephos; 2) microbial bacterial toxin, *Bti*; 3) hormonal insect growth regulators, pyriproxyfen (PPF); 4) the chitin biosynthesis inhibitor, hexaflumuron; following WHO guidelines [32]. Industrial grade temephos (87.4%) and PPF (98.3%) were provided by the Chinese Centers for Disease Control and Prevention. *Bti* (7000 ITU/mg) was provided by Wuhan Nature’s Favour Bioengineering. Hexaflumuron (99.0%) was provided by Shanghai Yien. Twenty-five 3-4-instar *Ae*. *albopictus* larvae were added to 99 mL of dechlorinated tap water and 1 mL of different concentrations of insecticide solution. Nine concentration gradients for each insecticide were tested during the experiment, with concentrations ranging between 10% and 90% mortality, three replicates per concentration. For temephos and *Bti*, the number of dead larvae was counted 24 h after the experiment, and the LC_50_ was calculated. For PPF and hexaflumuron, emergence inhibition was measured daily until complete mortality or adult emergence, and IE_50_ was calculated.

### DNA extraction and *kdr*, *ace-1* mutation detection

Genomic DNA was extracted from individual mosquitoes using the Extract-N-Amp Tissue PCR Kit (Sigma Aldrich) following the manufacturer’s protocol. Extracted DNA was stored at 4°C or used immediately for PCR. For each insecticide, 48 surviving individuals and 20 dead individuals were used to extract genomic DNA for mutation detection of target genes for insecticide resistance. DNA was extracted from individual mosquito and this is only done for adults which tested by the standard WHO tube test. Samples exposed to deltamethrin, permethrin, and DDT were genotyped at the voltage-gated sodium channel (VGSC) gene to detect mutations within domains II, III and IV, following the protocol by Kasai et al., 2011 [33]. Samples exposed to bendiocarb were genotyped at the *ace-1* gene to detect mutations within G119 following the protocol by M. Weill et al., 2004 [34]. The details of the primers and PCR conditions are given in S2 Table. A total of 204 samples were sequenced for the *kdr* gene, and 68 samples were sequenced for the *ace-1* gene. The sequences were aligned and analyzed using BioEdit (http://www.mbio.ncsu.edu/BioEdit/bioedit.html).

### Survey of insecticide usage

A survey on the use of pesticides in public regions was carried out via questionnaires from March to September of 2017 in the Yuexiu, Tianhe, Baiyun and Conghua districts of Guangzhou. The survey sites were selected near the collection sites of the *Ae*. *albopictus* samples. The surveys targeted employees of Pest Control Organizations (PCOs) and the local street administrators responsible for mosquito control. The contents of the survey included the species targeted and the frequency of insecticides used to control adult and larval mosquitoes. Each site completed 30 questionnaires.

### Statistical analysis

LC_50_ and KDT_50_ were estimated using the log-probit models. For larvae bioassays, the resistant status was measured by the resistant ratio (RR_50_), i.e., the ratio of LC_50_ (or IE_50_) for the field population over LC_50_ (or IE_50_) for the laboratory-susceptible strain. Larval resistance status was defined as susceptible if RR_50_ < 5, moderately resistant if 5 < RR_50_ < 10, and highly resistant if RR_50_ > 10 [28]. Post hoc Tukey’s HSD test of analysis of variance (ANOVA) was used to compare differences in RR_50_ among different study sites. For adult bioassays, resistant status was defined by mortality rate: Resistant if mortality < 90%, probably resistant if mortality was between 90 and 98%, and susceptible if mortality > 98% [27, 28]. The relationship between nonsynonymous mutations and resistance was verified by Fisher's exact test or the χ^2^-test (when all n >5), and the odds ratio (OR) was calculated for each mutation. The χ^2^-test was used to compare differences in adult mortalities between deltamethrin and deltamethrin + PBO groups at different study sites.

## Results

### *Ae*. *albopictus* developed high resistance to currently used insecticides

**Fig 2:**
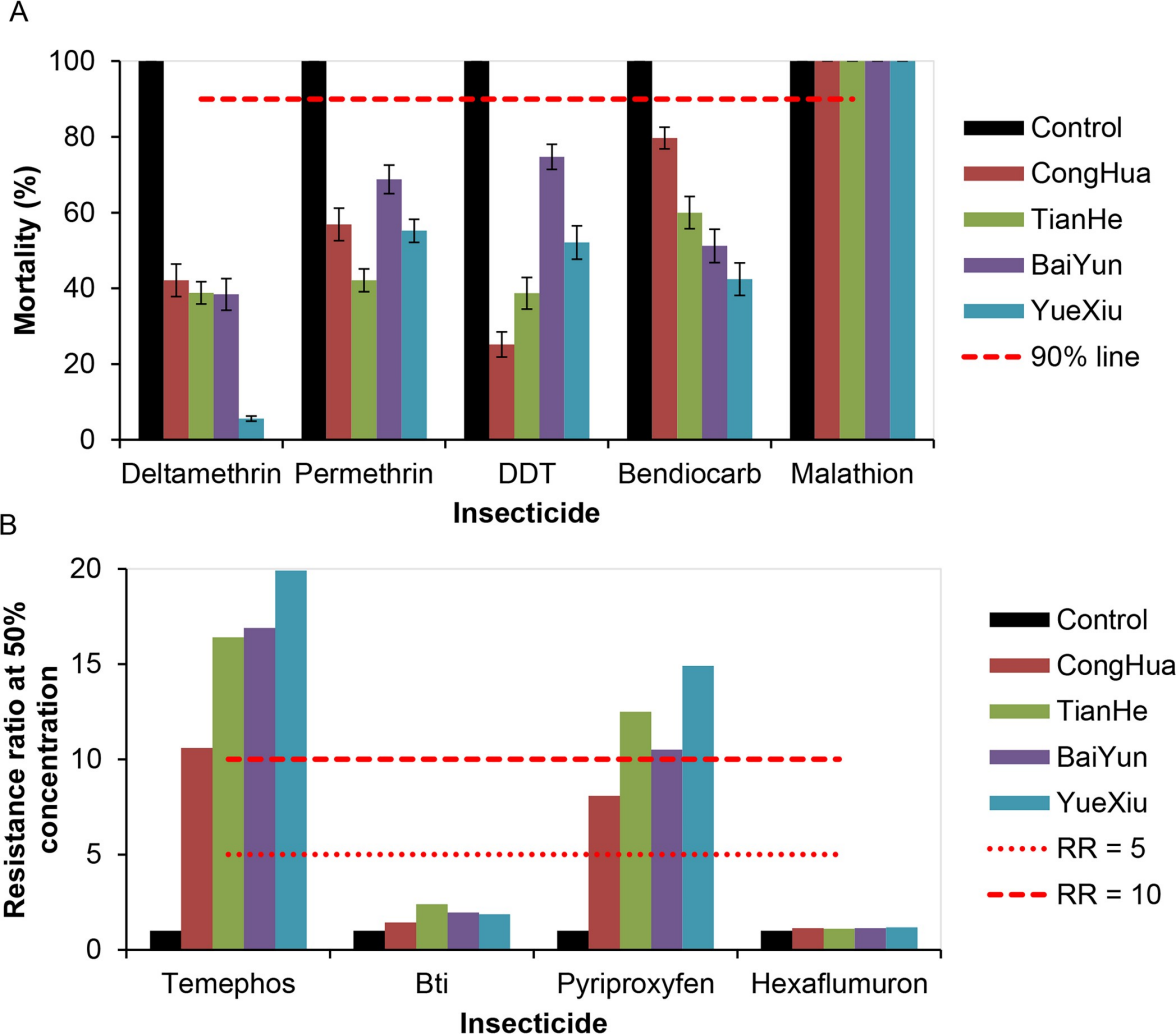
The resistance of *Aedes albopictus* to currently used insecticides. A: Resistance of adule *Aedes albopictus* to currently used insecticides, 2017. If the mortality is less than 90%, then the population is considered resistant. Error bars indicate 95% CIs. B: Resistance of larval *Aedes albopictus* to currently used insecticides, 2017. If the resistance ratio at 50% concentration (RR_50_) is <5, then the population is considered susceptible, when the RR_50_ is between 5 and 10, the population is considered to have moderate resistance, and when the RR_50_ is >10, the population is considered highly resistant.

*Ae*. *albopictus* adult populations in the four districts were all resistant to four insecticides (deltamethrin, permethrin, DDT and bendiocarb) (mortality<90%) except malathion (mortality >98%) (Fig 2A, S3 Table). The lowest mortality rate against deltamethrin and bendiocarb was 5.6% and 42.4% in the populations from Yuexiu, respectively, 42.1% to permethrin in Tianhe, and 25.2% to DDT in Conghua (Fig 2A, S3 Table). *Ae*. *albopictus* larvae from all four districts were still sensitive to *Bti* and hexaflumuron (RR_50_<5), but displayed high resistance to temephos and pyriproxyfen (RR_50_ > 10), and moderate resistance to pyriproxyfen (5 ≤ RR_50_ ≤ 10) in the Conghua population (Fig 2B, S4 Table). Comparatively, resistance to temephos and PPF were significantly higher in the urban areas, i.e., Yuexiu, Tianhe and Baiyun, than in the rural areas, Conghua.

### *Ae*. *albopictus* insecticide resistance may develop very fast

**Fig 3:**
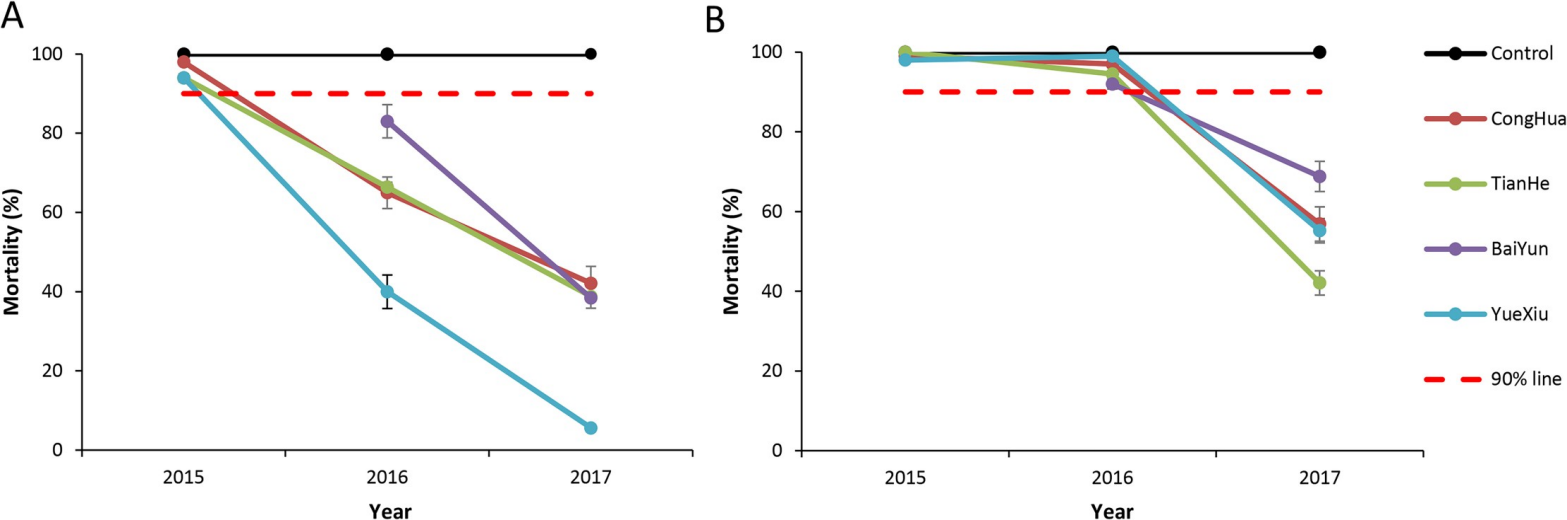
Resistance of *Aedes albopictus* to pyrethroid insecticides, 2015–2017. A: Resistance of *Aedes albopictus* to deltamethrin, 2015–2017. If mortality less than 90%, the population is considered resistant. Error bars indicate 95% CIs .B: Resistance of *Aedes albopictus* to permethrin, 2015–2017. If mortality less than 90%, the population is considered resistant. Error bars indicate 95% CIs.

Pyrethroids are the most commonly used insecticides to control the adult *Ae*. *albopictus* in Guangzhou. In 2015, *Ae*. *albopictus* populations was susceptible to deltamethrin in Conghua and possible resistant in Tianhe and Yuexiu (Fig 3A). In 2016, *Ae*. *albopictus* populations in all four districts became resistant to deltamethrin (Fig 3A). In 2017, *Ae*. *albopictus* mortality against deltamethrin was significantly decreased at all study sites compared to 2016, and the mortality in Yuexiu was only 5.6% (Fig 3A). The average mortality against deltamethrin was 95.3% in 2015. It decreased to 63.6% in 2016 and dropped again to 31.2% in 2017 (ANOVA, all *p* < 0.05), with a 30% decrease every year (Fig 3A). The decrease in *Ae*. *albopictus* mortality against permethrin was also very fast from 2016 (average 95.6%) to 2017 (55.8%), with a 40% decrease in one year (Fig 3B).

### Resistance to pyrethroids and DDT associated with 1534 codon mutations

Sequences of domains II (480 bp), III (346 bp) and IV (280 bp) of the VGSC gene were obtained from resistant and susceptible mosquitoes after deltamethrin, permethrin and DDT adult bioassays. Three synonymous mutations in domain II, 5 synonymous mutations in domain III, and 3 synonymous mutations in domain IV were detected (S5 Table). In domain III, non-synonymous mutations were detected at codon 1534, where wild-type TTC (Phe) was changed to either TCC (Ser) or CTC (Leu). Data analysis showed that the F1534S and F1534L mutations were significantly associated with the resistance to deltamethrin, permethrin and DDT (*p*<0.05) (Table 1).

**Table 1:** *Kdr* mutations at position F1534 of the VGSC gene in *Aedes albopictus*.

Insecticide	Phenotype	N	Genotype					Odds Ratio (95% CI)	
			FF	FS	SS	FL	LL	F1534S	F1534L
**Deltamethrin**	S	20	17	3	0	0	0	1	1
	R	41	2	1	17	3	18	4.44 (1.12, 17.52)*	NA **
**Permethrin**	S	19	11	5	0	1	1	1	1
	R	42	1	4	22	5	11	4.55 (1.38, 15.05) *	27.20 (4.60, 160.69) **
**DDT**	S	18	15	2	1	0	0	1	1
	R	44	5	7	15	11	6	5.00 (1.27, 19.74)*	NA **

NA: Not Available. Significance level

*p<0.05

**p<0.01

The 194-bp fragment in exon 5 of the AChE gene was obtained for *ace-1* mutation detection, but no amino acid mutation was found in the G119 site in the 33 successfully sequenced samples.

### PBO significantly reduced resistance to deltamethrin

**Fig 4:**
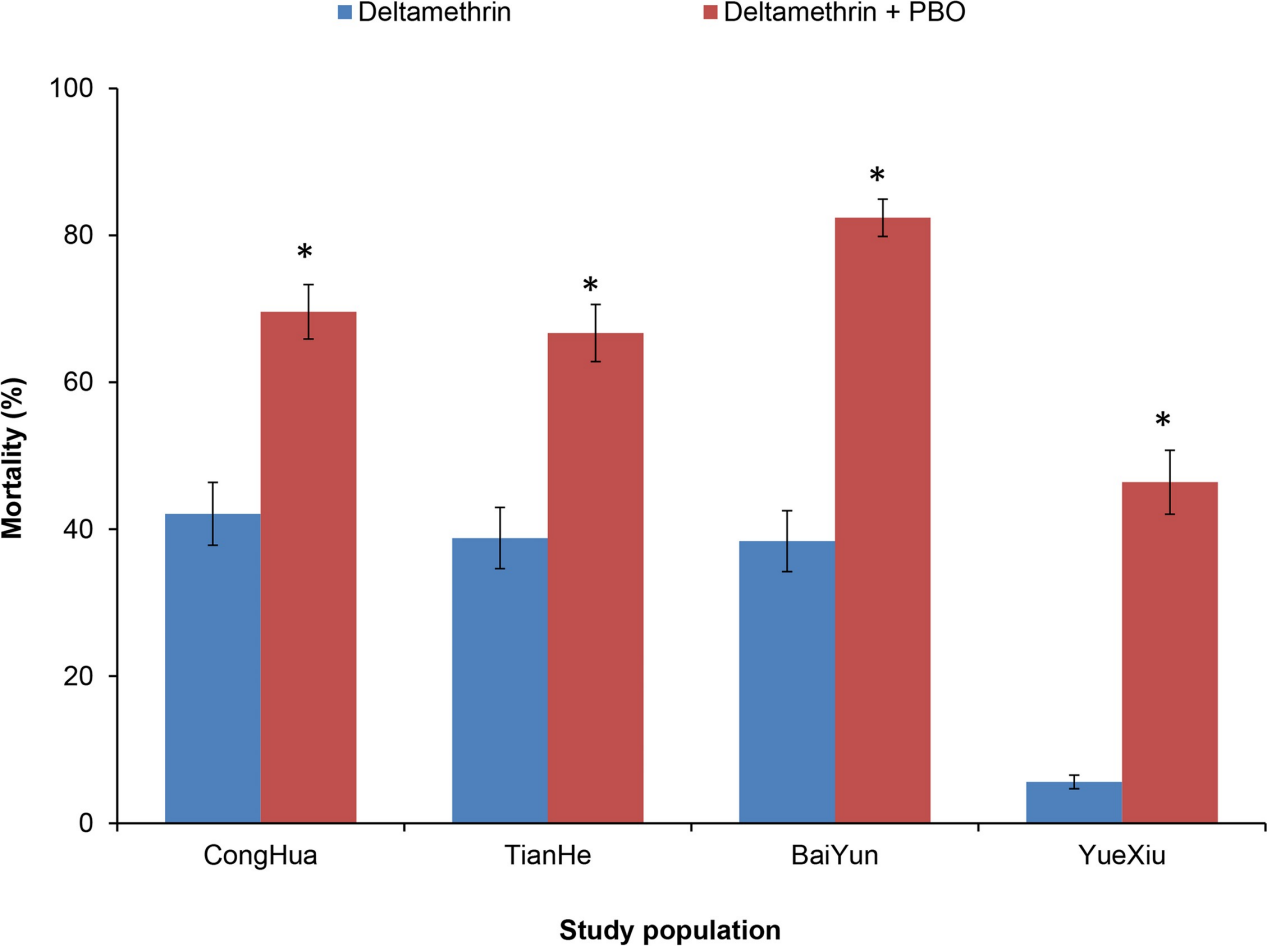
The effect of PBO on the resistance of *Aedes albopictus* to deltamethrin. Exposing mosquitoes to PBO before exposing them to deltamethrin significantly increased *Aedes albopictus* mortality compared to directly exposing them to deltamethrin. Error bars indicate 95% CIs. *: *p* < 0.01.

Exposing mosquitoes to PBO before exposing them to deltamethrin significantly increased *Ae*. *albopictus* mortality compared to directly exposing them to deltamethrin (χ^2^-test, all *p* < 0.01) (Fig 4), indicating that PBO can reduce the resistance of *Ae*. *albopictus* to deltamethrin through anti-P450s activity.

### Resistance to PPF may associated with cross resistance to deltamethrin

PPF is an insect growth regulator and has never been used previously in mosquito control in Guangzhou. However, resistance to PPF was detected in *Ae*. *albopictus* collected in all four study sites (Fig 2B). Comparative analysis on *Ae*. *albopictus* larval resistance to deltamethrin from four field populations and one laboratory selected resistant strain, R24, showed that all of them were resistant to deltamethrin as well as PPF (Table 2). Because R24 resistance to deltamethrin was artificially selected by exposing fully susceptible laboratory *Ae*. *albopictus* larvae to deltamethrin after 24 generations, this strain has never been exposed to any other insecticide; therefore, *Ae*. *albopictus* resistance to PPF could be explained by cross resistance to pyrethroids.

**Table 2:** Resistance to PPF and deltamethrin in *Aedes albopictus* from different populations, 2017.

Population name	Deltamethrin		pyriproxyfen	
	LC_50_ (95%CI) (mg/L)	RR_50_^b^	IE_50_ (95%CI) (μg/L)	RR_50_
**Control**^**a**^	0.001 (0.001, 0.001)	1.00	0.073 (0.064, 0.083)	1.00
**Yuexiu**	0.067 (0.061, 0.094)	67.0	1.091 (1.053, 1.142)	14.9
**Tianhe**	0.036 (0.035, 0.038)	36.0	0.913 (0.839, 1.062)	12.5
**Baiyun**	0.049 (0.046, 0.053)	49.0	0.767 (0.625, 0.806)	10.5
**Conghua**	0.037 (0.032, 0.040)	37.0	0.590 (0.555, 0.627)	8.08
**R24**^**c**^	0.033 (0.030, 0.036)	33.0	0.774 (0.663, 0.862)	10.6

^a^ Control: Laboratory-susceptible strain

^b^ RR_50_: resistant ratio, LC_50_ (or IE_50_) test population/LC_50_ (or IE_50_) laboratory-susceptible strain

^c^ R24: Laboratory-susceptible strains selected for 24 generations by deltamethrin

### Resistance and insecticide application

Our survey found that four major classes of insecticides (pyrethroids, organophosphates, organochlorine and carbamate) were currently used in Guangzhou (Table 3). Pyrethroids (mainly type I permethrin S-biomethrin, and type II beta-cypermethrin) were the most commonly used adulticides, while organophosphates (mainly temephos and fenthion) were the most commonly used larvicides (Table 3). The frequency of insecticide usage in urban areas was more frequent than that in the rural district of Conghua (Table 3). Conghua in the rural area did not use the insecticide routinely. The survey also found that *Bti* and nicotine (imidacloprid) have gradually become the new choices for larvae control (Table 3). Adulticides were more frequently used than larvicides.

**Table 3:** Survey of insecticide usage in four districts of Guangzhou, China, 2017.

District	N	Adulticide	Frequency	Larvicide	Frequency
**Conghua**	30	Pyrethrin: Beta-cypermethrin, Cypermethrin, S-bioallethrin	None or 1time/year	Organophosphate: Fenthion	When necessary ^a^
**Tianhe**	30	Pyrethrin: Permethrin, Meperfluthrin, Alphacypermethrin, S-bioallethrin	1–2 times/month	Organophosphate:Temephos; Microbial bacterial toxins: *Bti*; Nicotine: Imidacloprid	When necessary
**Baiyun**	30	Pyrethrin: Permethrin, Meperfluthrin, Alphacypermethrin, Beta-cypermethrin; Organophosphate: DDVP; Carbamate: Propoxur	2–3 times/month	Organophosphate: Fenthion Nicotine: Imidacloprid	When necessary
**Yuexiu**	30	Pyrethrin: Permethrin, Meperfluthrin, Alphacypermethrin, Beta-cypermethrin, Tefluthrin, S-bioallethrin; Organophosphate: DDVP; Carbamate: Propoxur	1–2 times/month, 2–3 times/week when an outbreak occurs	Organophosphate: Temephos, Fenthion; Microbial bacterial toxin: *Bti*; Carbamate: Fenobucarb; Nicotine: Imidacloprid	When necessary

^a^ When necessary: used during outbreaks

## Discussion

In this study, we characterized the current insecticide resistance in *Ae*. *albopictus* in Guangzhou, China in the following ways: (1) Increasing. In 2017, *Ae*. *albopictus* populations in four districts were all resistant to the four tested insecticides with sensitivity only to malathion (Fig 2), whereas in 2014, all were susceptibility but had only low or moderate resistance to some insecticide in very limited areas of Guangzhou [18, 35]. In 2017, all four tested districts were resistant (Fig 2). (2) Rapid. The resistance of *Ae*. *albopictus* to pyrethroids has changed from susceptibility or moderately resistant to resistant within three years, 2015–2017 (Fig 3). (3) Multiresistance. In 2014, *Ae*. *albopictus* was resistant only to limited pyrethroids and DDT, whereas in 2017, it was resistant to all four types of insecticides except malathion[11,19]. In 2016–2017, the Mosquito and Oviposition Positive Index (MOI) in Guangzhou is greater than 5 (MOI>5 is the risk of dengue virus transmission) [36]. At the same time, in 2015, dengue fever affected 31 towns in 8 districts in Guangzhou and 108 cases were reported; in 2016, dengue fever expanded to 61 towns in 10 districts, and 253 cases were reported; in 2017, dengue fever expanded to 122 towns in 11 districts and cases increased to 950, only less than 2013 and 2014 in the past 10 years [6].The quick generation, wide distribution, and increasing insecticide resistance to multiple agents in *Ae*. *albopictus* is bound to affect mosquito management and threaten the prevention and control of dengue, similar to the resistance in *Anopheles* mosquitoes has prevented the elimination of malaria [37,38].

Mutations in the VGSC gene have been correlated with the resistance of vector mosquitoes including *Ae*. *albopictus* [33,39,40]. The present study also found that F1534S and F1534L mutations were significantly correlated with the resistance of *Ae*. *albopictus* to deltamethrin, permethrin and DDT (*p*<0.05) (Table 1). At present, pyrethroids are the most commonly used insecticides in China; therefore, sensitive and specific techniques based on the detection of 1534 mutations must be developed to monitor the resistance of *Ae*. *albopictus* to pyrethroids. However, the mechanism for insecticide resistance in mosquitoes is very complicated. In addition to the correlation of *kdr* mutations with resistance, the increased expression and enhanced activity of P450s have also been proven to be associated with mosquito resistance to pyrethroids in the present study and in other studies [41–43]. Only P450s changes have been reported in the resistant mosquitoes, and no *kdr* mutations were reported [44]. Therefore, clarifying the mechanism of insecticide resistance in vector mosquitoes and developing suitable monitoring systems, especially those easily used in the field, remain challenging.

In the era of rapidly emerging and widely distributed insecticide resistance, it is important to make suitable and updated guidelines for insecticide usage. PBO is an inhibitor of monooxygenase, such as P450s [26,45]. The present study proved that PBO can significantly reduce the resistance of *Ae*. *albopictus* to deltamethrin by anti-P450s (Fig 4), which could be used as a synergistic agent to enhance the effect of pyrethroids. PPF is an insect growth regulator and has been used as an automatically disseminated insecticide to control habitats, especially for *Aedes* mosquitoes [46,47]. Recently, PPF has also been used to sterilize adult *Anopheles* mosquitoes by reducing their fecundity and longevity [48]. Unexpectedly, in the present study, the higher resistance to PPF was widely detected in *Ae*. *albopictus* populations in Guangzhou probably because of the cross resistance to pyrethroids (Fig 2B). Yunta C et al. also proved that PPF is metabolized by P450s and associated with pyrethroid resistance in *Anopheles gambiae* [41]. Considering the higher and widely distributed resistance to pyrethroids, they should be cautious when using PPF as the larvicide or as the synergistic agent for adulticide. In mosquito incense and aerosol insecticides used daily among residents, the active ingredient is primiarly pyrethroid (such as transfluthrin and s- bioallethrin). More and more residents are using mosquito nets, electric mosquito swatters or mosquito killer lamps to prevent mosquito bites. In Conghua, vegetable farmers use DDVP and beta-cypermethrin to prevent *Plutella xylostella*. According to our research, the *Ae*. *albopictus* populations were still sensitive to malathion, hexaflumuron and *Bti*. Similarly, in Brazil where pyrethroid and temephos resistance has developed, local health authorities recommend the use of malathion against adult mosquitoes and chitin synthesis inhibitors against larvae (Controle de vetores. http://www.saude.gov.br/vigilancia-em-saude/controle-de-vetores). Therefore, we suggest useing malathion against adult mosquitoes and hexaflumuron or *Bti* against larvae for dengue vector control in Guangzhou. Timely monitoring of resistance is critical for the proper management of insecticides. Additionally, every year at the end of February or early in March, the Guangzhou government launches a patriotic health campaign to focus on cleaning up aquatic mosquito habitats for one month, which is important for cleaning the over winter eggs of *Ae*. *albopictus* and reducing the population density of mosquitoes.

In conclusion, increased and more widespread insecticide resistance to multiple agents has been rapidly developing in *Ae*. *albopictus*, the primary dengue virus vector in China. Extensive applications and inapposite applications of insecticides were likely one reason for development of resistance generation. The 1534 codon mutations in the VGSC gene were significantly correlated with resistance to pyrethroids and possibly used as a biomarker to monitor insecticide resistance. PBO can significantly reduce the resistance of *Ae*. *albopictus* to deltamethrin an act as a synergistic agent of pyrethroids. Gravid *Ae*. *albopictus* does not oviposit all eggs into one place, although they used to lay eggs in different breeding sites. By treatment of a breeding site with an insecticide such pyriproxyfen (PPF), gravid mosquitoes could be contacted and contaminated with PPF when they oviposit the eggs. Then, when they fly to neighborhood breeding sites to lay the remaining eggs, the contaminated PPF would be transferred automatically to cryptic habitats [49, 50]. PPF may display cross resistance to deltamethrin, and the concentration should be cautiously considered when used as an automatically disseminated insecticide. Fast emerging resistance in *Ae*. *albopictus* raises the alarm for dengue vector control and calls for timely and guided insecticide management.

## Supporting information

S1 Table Detailed description of the geographic site of mosquito collection in Guangzhou. (DOCX)

S2 Table Primer and PCR conditions used for amplification and sequencing of the VGSC gene and the AChE gene of *Aedes albopictus* in Guangzhou. (DOCX)

S3 Table Knockdown time of the *Aedes albopictus* population from Guangzhou using the standard WHO tube susceptibility bioassay. ^a^Control: Laboratory-susceptible strain
(DOCX)

S4 Table Resistance bioassay results of larval *Aedes albopictus* in Guangzhou. ^a^Control: Laboratory-susceptible strain.
(DOCX)

S5 Table Prevalence of SNPs^a^ in the VGSC gene of *Aedes albopictus* in Guangzhou. ^a^ SNPs: Single nucleotide polymorphisms.
(DOCX)
