# Supplementary material for: Fast emerging insecticide resistance in Aedes albopictus in Guangzhou, China: Alarm to the dengue epidemic
Source: PLoS Negl Trop Dis. 2019 Sep 16;13(9):e0007665. doi: 10.1371/journal.pntd.0007665 (PMC6762209; doi:10.1371/journal.pntd.0007665)
Supplement: S1 Table — (DOCX) [file pntd.0007665.s001.docx]

**Table S1. Detailed description of the geographic site of mosquito collection in Guangzhou.**

| Geographical classification | District | Street or village | Coordinates | |
| --- | --- | --- | --- | --- |
|  |  |  | **Latitude (°N)** | **Longitude (°E)** |
| Old downtown | Yuexiu | Liurong sterrt | 23.073883 | 113.152797 |
|  |  | Pei Zheng Middle School | 23.071585 | 113.175161 |
|  |  | Kuangquan street | 23.094390 | 113.151586 |
| New downtown | Tianhe | Yuancun | 23.07506 | 113.224035 |
|  |  | Tianpingjia | 23.094204 | 113.190764 |
|  |  | Yuangang | 23.103406 | 113.202927 |
| Suburban | Baiyun | Southern Medical University | 23.111900 | 113.200371 |
|  |  | Jiahe | 23.140288 | 113.175798 |
|  |  | Jingxi street | 23.104833 | 113.192013 |
| Rural | Conghua | Lianxing village | 23.324505 | 113.361256 |
|  |  | Jiangpu | 23.323012 | 113.354751 |
|  |  | Xincunbei | 23.321474 | 113.360806 |
