# Supplementary material for: Fast emerging insecticide resistance in Aedes albopictus in Guangzhou, China: Alarm to the dengue epidemic
Source: PLoS Negl Trop Dis. 2019 Sep 16;13(9):e0007665. doi: 10.1371/journal.pntd.0007665 (PMC6762209; doi:10.1371/journal.pntd.0007665)
Supplement: S2 Table — (DOCX) [file pntd.0007665.s002.docx]

**Table S2. Primer and PCR conditions used for amplification and sequencing of the VGSC gene and the AChE gene of *Aedes albopictus* in Guangzhou.**

| **Gene** | **Location** | **PCR Primers (5’-3’)** | **PCR condition** |
| --- | --- | --- | --- |
| **VGSC** | Domains II | GACAATGTGGATCGCTTCCC | Initial denaturation at 94˚C for 3 min, 35 cycles each of 94˚C for 30 s, 55˚C for 30 s, and 72˚C for 45 s, followed by a final elongation step at 72˚C for 10 min |
|  |  | GCAATCTGGCTTGTTAACTTG |  |
|  | Domains III | GAGAACTCGCCGATGAACTT | Initial denaturation at 94˚C for 3 min, 35 cycles each of 94˚C for 30 s, 59˚C for 30 s, and 72˚C for 1min, followed by a final elongation step at 72˚C for 6 min |
|  |  | TAGCTTTCAGCGGCTTCTTC |  |
|  | Domains IV | TCGAGAAGTACTTCGTGTCG | Initial denaturation at 94˚C for 3 min, 35 cycles each of 94˚C for 30 s, 55˚C for 30 s, and 72˚C for 45 s, followed by a final elongation step at 72˚C for 10 min |
|  |  | AACAGCAGGATCATGCTCTG |  |
| **AchE** |  | CCGGGNGCSACYATGTGGAA | Initial denaturation at 94˚C for 3 min, 35 cycles each of 94˚C for 30 s, 52˚C for 30 s, and 72˚C for 1 min, followed by a final elongation step at 72˚C for 10 min |
|  |  | ACGATMACGTTCTCYTCCGA |  |
