# Supplementary material for: Fast emerging insecticide resistance in Aedes albopictus in Guangzhou, China: Alarm to the dengue epidemic
Source: PLoS Negl Trop Dis. 2019 Sep 16;13(9):e0007665. doi: 10.1371/journal.pntd.0007665 (PMC6762209; doi:10.1371/journal.pntd.0007665)
Supplement: S3 Table — aControl: Laboratory-susceptible strain (DOCX) [file pntd.0007665.s003.docx]

**Table S3. Knockdown time of the *Aedes albopictus* population from Guangzhou using the standard WHO tube susceptibility bioassay.**

| **Population**  **Name** | **Deltamethrin（0.03%）** | **Permethrin（0.25%）** | **DDT（4%）** | **Malathion（0.8%）** | **Bendiocarb（0.1%）** |
| --- | --- | --- | --- | --- | --- |
|  | KDT_50_ （95%CI）（min） | KDT_50_（95%CI）（min） | KDT_50_（95%CI）（min） | KDT_50_（95%CI）（min） | KDT_50_（95%CI）（min） |
| **Conghua** | 42（42，47） | 49（45，53） | 70（48，409） | 45（43，48） | 50（46，55） |
| **Tianhe** | 84（77，96） | 77（69，91） | 41（36，55） | 52（49，54） | 70（64，78） |
| **Baiyun** | 79（70，94） | 77（70，89） | 34（32，38） | 50（48，52） | 76（69，88） |
| **Yuexiu** | 91（81，107） | 85（72，101） | 79（55，101） | 55（53，58） | 75（68，87） |
| **Control^a^** | 25（21，28） | 22（20，24） | 26（24，27） | 33（30，37） | 32（29，35） |

^a^Control: Laboratory-susceptible strain
