# Supplementary material for: Fast emerging insecticide resistance in Aedes albopictus in Guangzhou, China: Alarm to the dengue epidemic
Source: PLoS Negl Trop Dis. 2019 Sep 16;13(9):e0007665. doi: 10.1371/journal.pntd.0007665 (PMC6762209; doi:10.1371/journal.pntd.0007665)
Supplement: S4 Table — aControl: Laboratory-susceptible strain. (DOCX) [file pntd.0007665.s004.docx]

**Table S4. Resistance bioassay results of larval *Aedes albopictus* in Guangzhou**

| **Population**  **Name** | **Temephos** | **Bti** | **Pyriproxyfen** | **Hexaflumuron** |
| --- | --- | --- | --- | --- |
|  | **LC_50_(95%CI)（μg/L）** | **LC_50_（95%CI）(mg/L）** | **IE_50_ （95%CI）（μg/L）** | **IE_50_（95%CI）（μg/L）** |
| **Conghua** | 16.0  （15.4，18.2） | 0.063  （0.056，0.073） | 0.590  （0.555，0.627） | 3.24  （3.16，3.44） |
| **Tianhe** | 24.7  （17.3，38.9） | 0.105  （0.092，0.125） | 0.913  （0.839，1.062） | 3.20  （3.04，3.37） |
| **Baiyun** | 25.5  （25.0，28.3） | 0.086  （0.042，0.139） | 0.767  （0.625，0.806） | 3.23  （3.06，3.44） |
| **Yuexiu** | 30.1  （28.0，32.0） | 0.083  （0.071，0.102） | 1.091  （1.053，1.142） | 3.38  （3.16，3.66） |
| **Control^a^** | 1.51  （1.42，1.62） | 0.044  （0.040,0.050） | 0.073  （0.064，0.083） | 2.87  （2.75，3.00） |

^a^Control: Laboratory-susceptible strain.
