# Supplementary material for: Fast emerging insecticide resistance in Aedes albopictus in Guangzhou, China: Alarm to the dengue epidemic
Source: PLoS Negl Trop Dis. 2019 Sep 16;13(9):e0007665. doi: 10.1371/journal.pntd.0007665 (PMC6762209; doi:10.1371/journal.pntd.0007665)
Supplement: S5 Table — a SNPs: Single nucleotide polymorphisms. (DOCX) [file pntd.0007665.s005.docx]

**Table S5. Prevalence of SNPs^a^ in the VGSC gene of *Aedes albopictus* in Guangzhou.**

| **Domains** | **SNPs** | | **Occurrence of mutations** | |
| --- | --- | --- | --- | --- |
|  | **Amino acids** | **Codon change** | **N** | **%** |
| **Ⅱ** | V945V | GTG>GTA | 190 | 100 |
|  | C947C | TGT>TGC | 190 | 100 |
|  | C957C | TGC>TGT | 72 | 37.9 |
| **Ⅲ** | L1480L | CTC>CTT | 54 | 29.3 |
|  | N1481N | AAC>AAT | 54 | 29.3 |
|  | E1493E | GAG>GAA | 6 | 3.3 |
|  | T1507T | ACA>ACG | 97 | 52.7 |
|  | E1508E | GAA>GAG | 97 | 52.7 |
| **Ⅳ** | A1691A | GCT>GCC | 180 | 100 |
|  | N1712N | AAT>AAC | 51 | 28.3 |
|  | F1713F | TTT>TTC | 32 | 17.8 |

^a^ SNPs: Single Nucleotide Polymorphisms.
